# Functional Impairment in Borderline Personality Disorder: The Mediating Role of Perceived Social Support

**DOI:** 10.3389/fpsyg.2022.883833

**Published:** 2022-05-27

**Authors:** Beatriz Thadani, Ana M. Pérez-García, José Bermúdez

**Affiliations:** Department of Personality, Assessment and Psychological Treatment, Faculty of Psychology, Universidad Nacional de Educación a Distancia, Madrid, Spain

**Keywords:** borderline personality disorder, personality traits, perceived social support, disability, functional impairment, personality disorders

## Abstract

Borderline personality disorder (BPD) is characterized by instability in relationships, mood fluctuations, and erratic behavior. This study investigates the relationship between pathological personality traits and functional disability, the status of perceived social support in BPD, as well as its mediating role in this relationship. In this cross-sectional study, 192 Spanish women (BPD group, *N* = 97; healthy control group, *N* = 95) completed, through two online platforms, a battery of tests including: the *Personality Inventory for DSM-5 Brief Form (PID-5-BF)*, the *World Health Organization Disability Assessment Schedule 2.0* (WHODAS 2.0) and the *Perceived Social Support subscale of the Quality of Life Questionna*ire (QLQ). The results show that perceived social support was significantly lower in the BPD group, which also presented a significantly higher disability score than the control group. Pathological personality traits affected functionality both directly and indirectly through perceived social support, as this variable was a significant mediator in both groups. We conclude that perceived social support is impaired in BPD patients, and enhancing it as a complementary therapy to evidence*-*based treatments could help preserve the functionality of patients while pathological traits are regulated. This study also encourages future research to delve into the relevance of other psychosocial variables on the functionality of subjects with BPD, and the need of enhancing them in therapy.

## Introduction

Borderline personality disorder (BPD) is a severe and debilitating psychiatric disorder classified in the Diagnostic and Statistical Manual of Mental Disorders (DSM-5) within Cluster B of personality disorders. It is characterized by unstable relationships, fear of abandonment, and an inability to regulate emotions (American Psychiatric Association [APA], 2013). In the United States, large epidemiologic studies have estimated the point prevalence of BPD in the general population at 1.7% (Gunderson et al., 2018), however the most worrying aspect of this condition is its presence in clinical settings. The prevalence of BPD in the psychiatric outpatient population has been estimated at 11%, and in the psychiatric inpatient population it is as high as 20% (American Psychiatric Association [APA], 2013). We find the same trend in Spain, where the prevalence of BPD in the general population is between 1% and 2%, while in outpatients and inpatients it is between 11% and 20% and 18% and 32%, respectively (García-López et al., 2010). This highlights that BPD is a disorder where patient’s symptoms tend to be so overwhelming that they feel strongly compelled to seek help and treatment. This could be considered an indicator of how disruptive BPD can be and how disruptive it is in patients’ lives and functioning.

A meta-analytic review on the long-term course of BPD in clinical adult populations has found that between 50% and 70% of BPD patients achieve remission in the long term; nevertheless, findings suggest that the improvement tends toward symptomatic amelioration and that only slight functional improvements are seen in the long term (Álvarez-Tomás et al., 2019). In general, a large number of BPD traits correlate inversely with age, with the most pronounced decline in symptoms occurring after 44 years of age (Grant et al., 2008); however, although symptoms decrease, severe social dysfunction persists as well as problems in maintaining steady employment (Gunderson et al., 2011). This suggests that the expression of the disorder varies with age, but that the underlying severity and distress remains unaltered. Even so, we must emphasize that the natural course of BPD is characterized by its plasticity, with consecutive periods of remission and relapse (Álvarez-Tomás et al., 2019), and as a result the patient will exhibit different manifestations according to the current state of the disorder. It is therefore important to recognize what factors are relevant in this remission–relapse cycle other than BPD patient’s tendency to show improvement over time. Research suggests that older patients not only maintain the functional impairment that they had in their youth, but they may also have worse functionality than younger patients across physical and psychological domains (Frías et al., 2017). Maintaining optimal levels of functionality for BPD patients could then be an ongoing process that requires that we are aware of the factors involved in order to help them more effectively.

The concept of functional disability refers to disorders’ impact on daily functioning. The World Health Organization (WHO) has developed a model for health called the International Classification of Functioning, Disability and Health (ICF). This lack of functionality occurs within a specific context and the degree of disability in an individual is a dynamic interaction between their disease and contextual factors, which takes into account how the social, attitudinal, and physical world can impact and be impacted by the individual’s condition (World Health Organization [WHO], 2001).

Like for all personality disorders, a BPD diagnosis requires that patients have difficulties that are pervasive, inflexible, impairing, and outside the norm for their cultural background (American Psychiatric Association [APA], 2013). BPD traits may be especially disabling, as their functional limitations to engage in work or leisure activities, as well as in social relationships, are even more pronounced than those commonly associated with other personality disorders or depressive disorders (Skodol et al., 2002). Also, attaining and maintaining sustained remissions or recoveries, that involve not only symptomatic remission but good social and vocational functioning, is substantially more difficult for BPD patients than for those with other types of personality disorders (Zanarini et al., 2012). Research has also found that compared to healthy controls, their functioning is lower and BPD patients fail to maintain stable employment or at least one healthy relationship (Javaras et al., 2017), and their symptoms are consistently associated with impaired work performance (Juurlink et al., 2018). The main controlling variables and the reasons why their level of impairment is so remarkable, even compared to other severe mental disorders, are still unclear. To unravel this, we must understand how personality traits relate to the ability to function in all the areas of life and analyze possible intermediate factors, especially those factors related to positive psychology and strengths, as they have not yet received sufficient attention in relation to BPD. There is a pressing need to go deeper into this matter because this condition can be so detrimental for patients that borderline personality can be considered as a marker of serious health problems across a person’s life span (Botter et al., 2021).

Social support, especially perceived social support, could play a critical role in the level of disability experienced by a person with BPD. Perceived social support refers to support individuals view as readily available and suitable during stressful periods; it is therefore a subjective view (Pierce et al., 1991), while received social support is the actual, objective support received during those periods of need. These two dimensions are not interchangeable and may affect the individual in different ways. Our interest in this variable lies in the fact that perceived support is consistently associated with positive health outcomes and, consequently, functionality (Uchino et al., 2012).

Numerous research studies have confirmed the important role of perceived social support in the functionality of different populations. It appears to be a valuable protective mechanism that can not only protect but also improve psychological well-being by maintaining positive emotions and mitigating stress (Thoits, 2011). The relationship between high social support and optimal mental and physical health has been observed in a variety of populations, including college students, workers, the unemployed, mothers, widows, and parents of children with serious medical conditions (Ozbay et al., 2007). Socially integrated people tend to be healthier, both physically and mentally, than those who are socially isolated (Uchino et al., 2012). Elements associated with perceived social support, such as perceived availability of a confidant and satisfaction with their support mediate the effects of disability caused by depressive symptoms in later life (Yang, 2006). Also, when confronted with a physical condition, such as chronic pain, higher levels of perceived social support predict improvements in patients’ quality of life, disability, depression, and anxiety (Van Dyke et al., 2018).

Research on the characteristics of BPD patients’ relationships indicate that they might exhibit low levels of objective and perceived social support, which means they are probably unable to enjoy the benefits of this protective variable on their health and their functionality. As numerous studies have indicated, relational problems could be the cornerstone of BPD (Stanley and Antonia, 2017). The dysfunctionality and instability of their relationships are consistent with some of the most significant traits of the disorder, such as perceiving others from a perspective of either idealization or devaluation, extreme fears of abandonment, and exaggerated attempts to avoid such abandonment, sometimes through angry or hostile behavior (American Psychiatric Association [APA], 2013). The poor quality of their relationships may also be due to factors such as their fearful and preoccupied attachment styles compared to controls (Hashworth et al., 2021) and their sensitivity to rejection. This tendency to expect and anxiously perceive rejection may amplify fears of loss and abandonment, as well as the impulsivity and anger experienced in this disorder, usually in response to those fears. These cognitions and behaviors can erode both objective social support and perceptions of social support, and ultimately satisfaction in their relationships (Lazarus et al., 2016). It is also worth noting that the structure of their social networks is characterized by people who do not bring them stable and frequent relationship satisfaction (Lazarus et al., 2020). BPD patients also manifest a bias in their detection of social cues because they tend to judge others’ faces as unapproachable and untrustworthy (Nicol et al., 2013). Thus, people with BPD are habitually exposed to negative stimuli and have difficulty in recognizing a positive emotional state in others (Kleindienst et al., 2019) and are themselves afraid of being positively evaluated by others (Weinbrecht et al., 2020). These vulnerabilities in the detection and perception of social indicators may translate into a low perception of social support by not seeing others as allies but as threats. Regarding their social networks from a wider perspective, they seem to have poorer social integration and, therefore, do not receive as much as healthy individuals the benefits the community can provide like a sense of belongingness and support (Beeney et al., 2018). To our knowledge, perceived social support in BPD has barely been studied. To examine this variable in BPD patients can provide a better understanding of their compromised relationships and shed light on the factors that could be involved in their low functionality, which could be due to underdeveloped strengths, such as perceived social support.

The aim of this study was to analyze functional disability in BPD and its relationship with personality traits and perceived social support. We were able to define the state of functionality and perceived social support in patients with this disorder and propose a significant preliminary model of functional impairment in BPD.

Considering our aim as well as previous literature, we hypothesized the following: (1) higher pathological personality traits will correlate with higher functional impairment, therefore the BPD group will show a higher disability score across all life domains than the control group; and (2) the BPD group will have a lower score in perceived social support than the control group, and this variable will also be a significant mediator in the relationship between personality traits and functionality in both groups.

## Materials and Methods

### Participants

A total of 237 women participated in this study: 130 in the control group and 107 in the clinical group. Ten participants were excluded from the BPD group as they had not been diagnosed by a mental health professional at the time of research. In addition, 35 women were excluded from the control group because they had not completed all the tests, exceeded the maximum age, or had not reached the minimum perceived mental and physical state required to participate in the research.

The final sample thus consisted of 192 women: 97 in the clinical group and 95 in the control group. The common inclusion criteria for both groups were that they had to reside in Spain and be of Spanish nationality in order to control as far as possible cultural and environmental differences. The participants also had to be between 18 and 55 years of age in order to control the possible deterioration detected in functionality was not due to age-related problems. In the clinical group, participants had to have a firm BPD diagnosis received by a psychiatrist and conducted on the basis of the DSM-5 criteria. Participants in the control group had to report a perceived mental and physical health score of at least 3 out of 5 (1 = poor, 2 = fair, 3 = good, 4 = very good, and 5 = excellent), not suffer from any physical or mental illness, and not have been in psychological therapy or taken psychiatric medication for at least one year. These criteria were designed to ensure that the women in the control group were in a good enough physical and mental state at the time of the study.

The BPD sample was obtained with the collaboration of three organizations from different parts of Spain dedicated to providing support for patients with personality disorders. They invited their members, as well as the users of their online groups, to participate in this research by explaining to them the objectives of the study and directing them to the study’s webpage for further information. The healthy control sample was composed of students of a Spanish distance and online university, the *Universidad Nacional de Educación a Distancia* (*UNED)*. The characteristics of this university allowed as to include participants with a wide range of ages, occupation and location in the country.

The mean age of the clinical/BPD group was 33 years (*SD* = 7.89). More than half of them (57.70%) were not married or in a stable relationship and 41.3% were neither working nor studying at the time of the research. In addition, 65.9% had been diagnosed with BPD for five years or less, 58.8% were diagnosed by public health services, 67.7% were taking prescribed medication for their disorder, and 71.1% were receiving psychotherapy at the time of the study.

The mean age of the control group was also 33 years (*SD* = 7.70). Among them, 31.6% were not married or in a stable relationship and 65.3% had a job (or other occupation) in addition to being a university student. In the control group, 86.3% had never taken medication for a mental health condition and 70.5% had never undertaken any form of psychotherapy. All the participants in the control group reported good perceived physical and mental health (at least 3/5), 63.2% described their physical health as very good (4/5) and 48.5% described their mental health as very good (4/5).

### Measures

The *Personality Inventory for DSM-5 Brief Form* (PID-5-BF) (American Psychiatric Association [APA], 2013) was used to assess participants’ personality traits. This instrument measures traits as having two opposing poles and assumes that there is continuity between the adaptive or resilient pole and the pathological or maladaptive pole. Traits, according to this scale, can present some adjustments across the life span —either due to maturity or as a result of experiences—and are not fixed or static characteristics (Torres-Soto et al., 2019). We used the Spanish short version of this measurement instrument, which consists of 25 items with five items in each of the following trait domains: “negative affectivity,” “detachment,” “disinhibition,” “psychoticism,” and “antagonism.” The items are rated on a 4-point Likert scale. The PID-5 scores are expressed as the mean score for the total scale as well as for each domain and range from 0 to 3. Higher mean scores indicate greater personality dysfunction. The abbreviated version (PID-5-BF) has correct reliability and factor structure (Anderson et al., 2018). In our sample it showed good internal consistency (α = .96).

The *World Health Organization Disability Assessment Schedule 2.0* (WHODAS 2.0) (American Psychiatric Association [APA], 2013) assesses functionality and disability by adopting the International Classification of Functioning, Disability and Health (ICF) as a reference (World Health Organization [WHO], 2001). It is a standard instrument to compare different populations. It measures health, disability, and limitations based on participants’ experiences over the last 30 days, by assessing 36 items in six categories: “understanding and communication,” “ability to move around,” “self-care,” “getting along with others,” “activities of daily living,” and “participation in society.” Items are evaluated on a 5-point Likert scale (0 = no difficulty, 1 = mild, 2 = moderate, 3 = severe, and 4 = extreme).

In our research, we did not consider the items related to studies or work in the domain “activities of daily living,” since the consequences of characteristics of the disorder meant that many of the participants were not engaged in any academic or work activities at the time of the investigation.

As for the way of scoring, the total score varied from 0 to 100, where higher scores indicate greater disability. The scale allows the score to be calculated in a simple and complex way. In our study we opted for the complex way of scoring, namely, item response theory-based scoring (IRT), which allows for a more detailed analysis and is more useful when comparing responses between populations.

The psychometric properties of the 36-item Spanish version performs well when applied to community and clinical populations and is able to differentiate between patients with different intensities of clinical symptoms (Guilera et al., 2015). In our sample, and after excluding the items mentioned, the WHODAS 2.0 has a high internal consistency reaching a Cronbach’s alpha of.97.

The *Perceived Social Support subscale of the Quality of Life Questionna*ire (QLQ) (Ruiz and Baca, 1993) is a self-administered 5-point Likert scale with participants having to rate each item (1 = not at all, and 5 = very much). The questions assess participants’ current social situation based on various aspects related to daily life and take into account elements like feelings of social connection and belonging and satisfaction with relationships with family and friends.

It is composed of nine items in the case of individuals not in a romantic relationship at the time of answering the questionnaire and 13 items for those who are in a romantic relationship. In our study, we excluded the four additional items as well as the item on sexual relationships. We decided to do this since our sample was diverse in relation to marital status—unstable and stormy relationships are also a hallmark of BPD, as well as impulsivity in sexual relationships. We considered it more appropriate to measure dimensions of perceived social support that are not a direct part of the symptomatology of this disorder. Therefore, the scale used in this study was composed of eight items. After excluding these items, we found the scale still had a good internal consistency (α = .93) in our sample.

### Procedure

In this cross-sectional study, participants completed the battery of tests in one session through two online platforms—one for the control group and one for the clinical group. Each platform’s home page explained the study’s objectives and requirements, and how to participate. After providing a written informed consent participants completed a registration form that gave them access to the battery of tests. A contact section was also enabled for additional questions about the study or questionnaire so that we could provide the greatest possible amount of assistance to both study groups. These measures were taken to ensure the tests were completed correctly, to correct any possible errors in understanding or approach in order to obtain reliable results and conclusions and to alleviate, as far as possible, the shortcomings of a participation through digital media.

### Data Analysis

Statistical analyses were performed using SPSS software (IBM SPSS Version 22.0). Differences between the clinical and control groups were analyzed using the *χ^2^* test for the sociodemographic variables and Student’s *t*-tests for the differences in psychological variables using 95% confidence intervals. Effect sizes were calculated using Cohen’s *d*. Linear correlations between the continuous variables were analyzed using Pearson’s correlation (*r*). PROCESS Macro Version 3.4 (Hayes, 2014) was used to perform the mediation analyses on each group and identify the total effect, the direct effect of personality on the dependent variable (functional impairment), and the indirect effect through perceived social support.

## Results

In relation to the sociodemographic variables, comparative analyses indicated that there was no difference in age between the two groups (*p* = .43), although there were more single (*χ^2^* = 18.91, *p* < .001, Cramer’s *V* = .31) and unemployed (*χ^2^* = 26.07, *p* < .001, Cramer’s *V* = .37) women in the clinical group.

Means, standard deviations, and Pearson’s correlations (*r*) were calculated for all the variables and for each group separately. In the clinical group, the personality domains of negative affectivity and disinhibition showed the strongest correlations with the different dimensions of the WHODAS 2.0, presenting significant values ranging from 0.31-0.52. In this group, perceived social support had a negative correlation with total personality, and specially with the domain of antagonism (–0.26). This variable also correlated negatively with disability, with significant values going from –0.22 to –0.36 (see Table 1). In the control group, the personality domain of negative affectivity also showed some of the strongest correlations with the WHODAS 2.0. scores, as well as detachment, the significant values ranged from 0.26 to 0.63. Perceived social support correlated negatively with pathological personality, especially with the domains of detachment (–0.48) and psychoticism (–0.42). This variable also correlated negatively with disability, the significant correlations with the domains of the WHODAS 2.0. ranged from –0.36 to –0.61 (see Table 2).

**TABLE 1:** Pearson correlation coefficients between all instruments analyzed in the clinical group (*N* = 97).

	PID-5-BF negative affectivity	PID-5-BF detachment	PID-5-BF antagonism	PID-5-BF disinhibition	PID-5-BF psychoticism	PID-5-BF total score	Perceived social support
WHODAS 2.0. Cognition	0.52**	0.41**	0.45	0.39**	0.46**	0.53**	–0.16
WHODAS 2.0. Mobility	0.46**	0.31**	0.19	0.32**	0.39**	0.48**	–0.31**
WHODAS 2.0. Self-care	0.40**	0.21*	0.15	0.33**	0.25*	0.38**	–0.27**
WHODAS 2.0 Getting along	0.45**	0.44**	0.26*	0.33**	0.38**	0.54**	–0.29**
WHODAS 2.0**.** Life activities	0.33**	0.20	0.24*	0.31**	0.15	0.34**	–0.27**
WHODAS 2.0. Participation	0.13	0.23*	0.22*	0.41**	0.16	0.33**	–0.22*
WHODAS 2.0. Total Score	0.53**	0.40**	0.26**	0.47**	0.40**	0.59**	–0.36**
Perceived social support	–0.16	–0.20*	–0.26**	–0.17	–0.14	–0.28**	−

*p < 0.05, ^**^p < 0.01.

**TABLE 2:** Pearson correlation coefficients between all instruments analyzed in the control group (*N* = 95).

	PID-5-BF negative affectivity	PID-5-BF detachment	PID-5-BF antagonism	PID-5-BF disinhibition	PID-5-BF psychoticism	PID-5-BF total score	Perceived social support
WHODAS 2.0. Cognition	0.57**	0.51**	0.31**	0.37**	0.54**	0.67**	–0.46**
WHODAS 2.0. Mobility	0.39**	0.34**	0.31**	0.08	0.27**	0.40**	–0.42**
WHODAS 2.0. Self-care	0.26*	0.35*	0.16	0.14	0.34**	0.34**	–0.47**
WHODAS 2.0 Getting along	0.57**	0.63**	0.35**	0.41**	0.57**	0.73**	–0.53**
WHODAS 2.0**.** Life activities	0.43**	0.49**	0.40**	0.34**	0.46**	0.60**	–0.36**
WHODAS 2.0. Participation	0.48**	0.41**	0.20	0.44**	0.49**	0.59**	–0.61**
WHODAS 2.0. Total Score	0.58**	0.48**	0.38**	0.40**	0.57**	0.72**	–0.58**
Perceived social support	–0.32**	–0.48**	–0.26*	–0.28**	–0.42**	–0.50**	−

*p < 0.05,^**^p < 0.01.

This indicated that greater dysfunction in personality correlated with greater functional impairment, while a higher level of perceived social support correlated with lower scores on disability and pathological personality traits.

In all the Student’s *t*-tests the differences were significant (*p* < .001) with very large effect sizes (*d* > .80). The clinical group reflected higher scores in pathological personality traits and disability than the control group, and lower scores in perceived social support (see Table 3).

**TABLE 3:** Descriptive statistics (mean and standard deviation), observed score ranges, Student’s *t*-tests results, and effect sizes (Cohen’s *d*).

	BPD group *M (SD)*	Control group *M (SD)*	Range	*t*	*d*
PID-5-BF Negative Affectivity	2.44(0.46)	1.24(0.65)	0–3	14.67***	2.13
PID-5-BF Detachment	1.70(0.64)	0.60(0.53)	0–3	12.76***	1.87
PID-5-BF Antagonism	1.54(0.61)	0.48(0.43)	0–2.80	13.87***	2.01
PID-5-BF Disinhibition	2.30(0.48)	0.69(0.56)	0–3	21.15***	3.09
PID-5-BF Psychoticism	1.92(0.68)	0.72(0.65)	0–3	12.53***	1.80
PID-5-BF Total Score	1.98(0.39)	0.75(0.40)	0.12–2.80	21.50***	3.11
WHODAS 2.0. Cognition	48.93(18.23)	10.13(12.42)	0–87.50	17.26***	2.49
WHODAS 2.0. Mobility	34.59(23.80)	4.89(10.21)	0–90	11.27***	1.62
WHODAS 2.0. Self-care	36.40(21.27)	4.14(7.33)	0–81.25	14.11***	2.03
WHODAS 2.0 Getting along	50.36(23.24)	15.53(20.39)	0–100	11.03***	1.59
WHODAS 2.0**.** Life activities	59.15(28.21)	11.78(17.84)	0–100	13.94***	2.01
WHODAS 2.0. Participation	61.79(17.17)	13.19(13.89)	0–96.88	21.58***	3.11
WHODAS 2.0. Total Score	48.54(16.02)	9.94(11.24)	0–81.25	19.36***	2.79
QLQ social support subscale	2.39(0.74)	4.14(.66)	1–5	17.43***	2.50

M, Mean, SD, Standard deviation, ^***^p < 0.001.

Mediation analyses were conducted using PROCESS Model 4 (Hayes, 2014). The indirect effects were tested with bias-corrected bootstrapping (*n* = 5,000) and 95% confidence intervals (*CI*) for the indices. The parameter was statistically significant when the 95% bootstrapped *CI* did not include zero.

As a first overview, we analyzed on both groups the effects of personality on disability considering the total score of the instruments. After, we proceed to conduct a detailed analysis considering the effect of each personality domain on each of the different dimensions of disability. We tested each of these models on each group individually and also on the whole sample.

**FIGURE 1:**
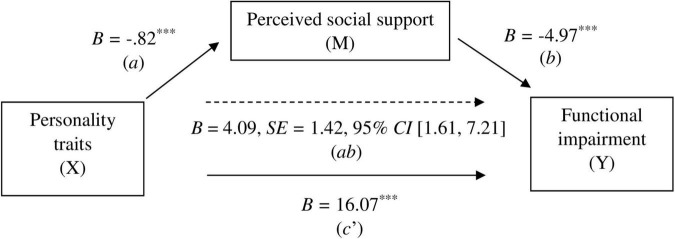
Mediation model results for the control group. *a* = effect of X on M, *b* = effect of M on Y, *ab* = indirect effect of X on Y through M, *c’* = direct effect of X on Y, *B* = unstandardized beta, *SE* = standard error, ^***^p < 0.001.

In the control group, the total effect (*c*) of personality traits on functional impairment was statistically significant (*B* = 20.16, *p* < .001). The direct effect was also significant (*B* = 16.07, *p* < .001), as well as the indirect effect on functionality via perceived social support: *B* = 4.09, *SE* = 1.42, 95% *CI* [1.61, 7.21] (see Figure 1). The proportion of the total effect explained by the mediator was 20.29% [*PM* = (indirect effect)/(total effect)].

**FIGURE 2:**
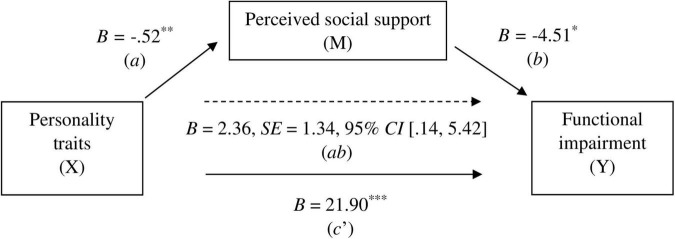
Mediation model results for the clinical group. *a* = effect of X on M, *b* = effect of M on Y, *ab* = indirect effect of X on Y through M, *c’* = direct effect of X on Y, *B* = unstandardized beta, *SE* = standard error, *p < 0.05, ^**^p < 0.01, ^***^p < 0.001.

In the clinical group, the total effect (*c*) of personality traits on functional impairment was statistically significant (*B* = 24.26, *p* < .001). The direct effect was also significant (*B* = 21.90, *p* < .001), as well as the indirect effect on functionality through perceived social support: *B* = 2.36, *SE* = 1.34, 95% *CI* [.14, 5.42]. The proportion of the total effect explained by the mediator was 9.73% (see Figure 2).

Considering each personality domain independently we found that, in the clinical group, the domains of detachment and antagonism were those that exerted an indirect effect through perceived social support on the disability scores of the patients. All personality domains, except antagonism, had significant direct effects on several disability dimensions, however the most relevant ones were disinhibition, which had a significant direct effect on all the WHODAS 2.0. dimensions, and negative affectivity, which showed significant direct effects on all domains of disability except on participation in society. On the other hand, in the control group, all personality domains, except negative affectivity, had a significant indirect effect on different dimensions of disability. All personality domains had significant direct effects on disability, however, the most relevant domains were negative affectivity, which exerted significant direct effects on all dimensions of the WHODAS 2.0. except on self-care, and disinhibition which had significant effects on all aspects of functionality, except for mobility and self-care. All significant indirect effects for both groups can be seen in Table 4.

**TABLE 4:** Mediation models with significant indirect effects in the clinical group and the control group.

	Indirect effect	Direct effect	Total effect
**Clinical Group**			
Detachment - Mobility	*B* = 1.91, *SE* = 1.25, [0.02, 4.83]	9.52**	11.44**
Antagonism- Mobility	*B* = 2.87, *SE* = 1.43, [0.44, 6.05]	4.54	7.41
Antagonism – Self-care	*B* = 2.27, *SE* = 1.27, [0.15, 5.11]	2.97	5.24
Antagonism – Getting along	*B* = 2.43, *SE* = 1.44, [0.13, 5.67]	4.71	9.89*
**Control Group**			
Detachment – Cognition	*B* = 3.06, *SE* = 1.09, [1.04, 5.33]	8.82***	11.88**
Detachment – Mobility	*B* = 3.06, *SE* = 1.79, [0.34, 7.29]	3.40	6.47***
Detachment – Self-care	*B* = 2.95, *SE* = 1.18, [0.87, 5.50]	0.52	3.47*
Detachment – Getting along	*B* = 5.46, *SE* = 2.22, [1.68, 10.37]	18.41***	23.88***
Detachment – Participation	*B* = 6.62, *SE* = 2.34, [2.87, 11.96]	4.17	10.79***
Antagonism – Cognition	*B* = 2.97, *SE* = 1.15, [1.12, 5.64]	5.95*	8.92**
Antagonism – Getting along	*B* = 5.68, *SE* = 2.44, [2.13, 11.60]	10.92*	16.60***
Antagonism – Life activities	*B* = 2.97, *SE* = 1.61, [0.60, 6.75]	13.44***	16.40***
Antagonism – Participation	*B* = 4.91, *SE* = 2.24, [1.65, 10.22]	1.32	6.23
Disinhibition – Cognition	*B* = 2.37, *SE* = 1.09, [0.53, 4.71]	5.85**	8.22***
Disinhibition – Getting along	*B* = 4.58, *SE* = 2.16, [0.85, 9.35]	10.26**	14.84***
Disinhibition – Life activities	*B* = 2.60, *SE* = 1.48, [0.29, 5.97]	8.19**	10.80***
Disinhibition - Participation	*B* = 3.63, *SE* = 1.86, [0.63, 7.82]	7.24***	10.87***
Psychoticism – Cognition	*B* = 2.21, *SE* = 0.68, [0.67, 3.37]	8.15***	10.37***
Psychoticism – Getting along	*B* = 4.64, *SE* = 1.92, [1.58, 9.02]	13.12***	17.76***
Psychoticism – Participation	*B* = 4.36, *SE* = 2.02, [1.34, 9.24]	5.99**	10.36***

Based on 5,000 bootstrap samples and 95% confidence intervals, *p < 0.05, ^**^p < 0.01, ^***^p < 0.001, B = unstandardized beta, SE = standard error.

When considering the whole sample, we found that our model of personality-disability was still valid and all personality domains had a significant direct effect and indirect effect, through perceived social support, on all the dimensions of the WHODAS 2.0. This information can be consulted on Supplementary Table 1.

The results indicate that in healthy individuals, as well as in BPD patients, personality affects functionality directly and indirectly, however the effect of personality via perceived social support is stronger in the control group.

## Discussion

The study assessed functionality and perceived social support in BPD women against a healthy control group, as well as the relationship between personality traits and functionality, and the mediating role of perceived social support. The results validated our hypotheses and we were able to draw the following conclusions.

First, the results show that functionality is highly impaired in BPD compared to the general population and that there is a direct correlation between a higher degree of pathological traits within the BPD spectrum and deteriorating functionality.

Specifically, the personality domains of negative affectivity and disinhibition showed the strongest correlations with disability, these are also the domains that have the highest presence in the BPD personality pattern according to our results and those of previous studies (Torres-Soto et al., 2019), since they presented the highest mean of all PID-5 subscales in this group. Their importance in BPD and their relationship with functionality indicate that they are two traits that should be addressed as a priority in order to restore some functionality in these patients. In the case of negative affect, this seems to be a particularly important trait, since it is also one of the domains that show the strongest correlations with functionality on healthy individuals.

It should be noted that the WHODAS 2.0 scores we obtained for the BPD sample not only show a lower total functioning score than the healthy control group, but their disability scores are also higher than those for other severe mental illnesses such as major depressive disorder, where patients present a WHODAS 2.0 score of 37.7 points (Chiang et al., 2021). BPD patients also seem to experience more disability than those with physical illnesses such as rheumatoid arthritis (score of 40.5 at baseline before treatment) (Meesters et al., 2010). While the scores of these two disorders show an impairment in daily functioning that could be considered as moderate, BPD patients’ scores are on the edge of severe disability with a mean score of 50 points out of a 100. Our findings support the fact that BPD appears to be as harmful to the dimensions related to health and functionality as diabetes, asthma, coronary heart disease, chronic pain, or Parkinson’s disease (Laurenssen et al., 2016).

Given the vast limitations BPD patients face daily, our study has tried to understand these challenges in as much detail as possible by examining them in the different domains of their lives and their link with personality. This could allow professionals to focus treatment on firstly restoring functionality in those aspects of daily living that are more closely related to pathological traits and affected by them, and therefore show a greater impairment.

Second, perceived social support presents at lower levels in BPD patients than in the general population, as shown in the differences between the two groups in this study. This could be because BPD patients perceive their relationships as more negative and more unstable than healthy people (Lazarus et al., 2020). This social instability and negativity can have serious consequences both for the individual and for their bonds with others, which in addition to being severe, only show modest improvement over the course of up to 16 years (Zanarini et al., 2010, 2012). As results, their social networks tend to become smaller and less significant over time compared to the general population (Lazarus and Cheavens, 2017). The few elements within their network are not viewed in a positive light, as they focus more on undesirable social feedback than healthy controls (Korn et al., 2016), a bias that may also have a negative impact on perceived social support. In addition, people with BPD, based on their social relationships, are at a disadvantage in this domain, as they create a close bond with people with whom they are, in reality, less connected within their own network (Beeney et al., 2018). This could increase their perception of a lack of available support as they seek it in bonds that are viewed as close only by the person with BPD.

All these interpersonal disturbances are reflected by the WHODAS 2.0 scores, that show that among the many functional problems experienced by BPD patients, the different social domains are where they experience the greatest disability.

As we can see, interpersonal dysfunction is a very prominent and discriminative feature in BPD that also seems to persist over time and after treatment. Therefore, it is probable that they will struggle to enjoy the benefits of perceived social support on health and functionality if this variable is not enhanced.

Last, perceived social support is a significant mediator in the relationship between personality traits and functionality, which leads us to conclude that in addition to the direct link between personality and functionality, the level of functional impairment is also affected indirectly through perceived social support. Therefore, traits affect functionality by two pathways, directly and indirectly, through this mediator.

Specifically, the personality domains of detachment and antagonism are the ones that acted indirectly on functionality, while psychoticism, disinhibition and, specially, negative affectivity acted directly on the WHODAS 2.0. scores. Therefore, the toxic effect of some traits could be controlled to some extent through intermediate variables and not just by acting on the trait itself. These two pathways appeared in both, the BPD sample and the control group; however, we found less significant mediations in the clinical group and this may be due to the ability of traits within the BPD spectrum to predict by themselves substantial impairment in quality of life (Perseius et al., 2006). Nevertheless, perceived social support remains a relevant intermediate variable in the acute impairment of these patients.

The significant role of perceived social support as a mediator was expected as earlier studies have already established a link between this variable and functionality. Adequate levels of social support can protect against the effects of diverse conditions and in various types of populations (Ozbay et al., 2007). Perceived social support is a resource that can reduce disabilities that are characteristic of different types of illnesses. In patients with chronic pain, high levels of perceived social support improve health-related quality of life and reduces functional impairment; it is therefore a variable that significantly affects the course of the disease (Van Dyke et al., 2018).

Psychiatric patients have low levels of social support, as well as a low quality of life. Moreover, both variables are also found to be related to each other, and therefore social support should be an essential part of psychiatric treatment due to its important role in improving the health and functionality of these patients (Gabal, 2017). In the case of BPD traits, negative interpersonal relationships, compared to the other characteristics of the disorder, are most strongly associated with a loss in quality of life through higher rates of disability, unemployment, and pain (Reynolds and Tragesser, 2019). The social domain is therefore key in this disorder and must therefore be enhanced.

The existence of two pathways by which personality acts on functionality suggests a need for a broader therapeutic approach—one that does not focus exclusively on addressing the maladaptive characteristics of the BPD personality pattern, but one that is also centered on the development of positive psychosocial and protective factors. A pathological personality is rigid and inflexible (American Psychiatric Association [APA], 2013), its treatment can be slow, and can require therapeutic assistance for long periods throughout a patient’s life. Perceived social support, like other variables of this nature, should be more flexible and respond faster to an intervention. An increase in perceived social support could in turn enhance the BPD patient’s level of resilience and ability to cope with problems and negative emotions (Nikmanesh and Honakzehi, 2016; Ghadirian et al., 2018; Cobo-Rendón et al., 2020). It could, therefore, be useful to help the BPD patient reframe their cognitions about their social network and teach them how to seek support more effectively, and at the same time work with families and significant others on more effective ways of conveying their support, which would also improve the overall quality of their relationships. Promoting healthy levels of psychosocial variables while treating the personality pathology, which is a lengthy process, could help patients preserve certain functionality and reduce the time needed for them to achieve “normal” levels, thus preventing the possibility of BPD patients experiencing chronic, residual impairment, and long-term deficits in functioning from which they could never really recover, even after they experience a symptomatic improvement (Skodol et al., 2005; Bozzatello et al., 2019).

This study has several limitations. First, it is focused on describing, proposing, and providing a preliminarily assessment of a model for functional impairment in BPD. It is an informative model on the role of traits and perceived social support in functionality. The importance of perceived social support will only be known in a more comprehensive way through methods that can prove causality, such as developing an intervention to enhance it and assessing the results of the intervention on patient functionality, and is reserved for future research. Also, although the analyzed mediation model is significant, there are still a large number of mediating variables that may be involved in the traits–functionality relationship, and future studies should delve into this, and discover the relevance of other psychosocial variables on the functionality of subjects with BPD, and the need of enhancing them in therapy. In order to achieve this, future research should take into account and analyze multiple intermediate variables of this nature in a more complex and comprehensive model of functionality in BPD, that also defines the causal relationships between the intervening variables. This will allow further investigations in this line of research to decide on the need to create treatment modules focused on developing psychosocial factors and strengths in BPD patients, and to know which of them to emphasize and prioritize.

Another limitation of this study is its cross-sectional nature, which has not allowed us to examine the evolution of traits, functionality, or the role of the mediator in BPD patients. This is especially relevant in a disorder such as BPD that undergoes significant changes throughout patient’s life (Biskin, 2015). Analyzing these variables in longitudinal studies or in research with different age groups should be considered in the future. Another limitation is that the sample consisted only of women. Therefore, future research should analyze these variables in men with BPD. Given the differences that exist in the manifestation of symptoms according to gender (Sher et al., 2019) it is logical to assume that there will also be differences in their pattern and degree of functional impairment, and the factors involved in it.

As a last limitation of our research, we would like to mention that by comparing the BPD group only with a healthy control group, we have not been able to find out if the dysfunctionalities of these patients are specific to this condition. In the future, it will be necessary to compare this disorder with other related illnesses, such as other personality disorders or bipolar disorder, in order to discover if the concrete functionality problems found and the role of perceived social support is specific to BPD.

Despite these limitations, we still have obtained valuable information about functional impairment in this disorder and the variables and possible mechanisms involved.

## Data Availability Statement

The raw data supporting the conclusions of this article will be made available by the authors, without undue reservation.

## Ethics Statement

The studies involving human participants were reviewed and approved by the Research Ethics Committee of the Universidad Nacional de Educación a Distancia. The patients/participants provided their written informed consent to participate in this study.

## Author Contributions

All authors provided their contribution to collection and analysis of data, preparation of manuscript, and approved the submitted version.

## Conflict of Interest

The authors declare that the research was conducted in the absence of any commercial or financial relationships that could be construed as a potential conflict of interest.

## Publisher’s Note

All claims expressed in this article are solely those of the authors and do not necessarily represent those of their affiliated organizations, or those of the publisher, the editors and the reviewers. Any product that may be evaluated in this article, or claim that may be made by its manufacturer, is not guaranteed or endorsed by the publisher.
