# Supplementary material for: Functional Impairment in Borderline Personality Disorder: The Mediating Role of Perceived Social Support
Source: Front Psychol. 2022 May 27;13:883833. doi: 10.3389/fpsyg.2022.883833 (PMC9197239; doi:10.3389/fpsyg.2022.883833)
Supplement: Supplementary file 1 [file Table_1.pdf]

**Supplementary Table 1. Direct, indirect, and total effects of all personality domains on the different dimensions of the WHODAS 2.0 in the whole sample (*N* = 192).**

| | Cognition | Mobility | Self-care | Getting along | Life activities | Participation |
|---|---|---|---|---|---|---|
| **Negative Affectivity** | | | | | | |
| Direct effect | 16.84*** | 11.48*** | 9.93*** | 16.44*** | 16.47*** | 12.63*** |
| Indirect effect | *B* = 6.99, *SE* = 1.25, [4.61, 9.45] | *B* = 7.33, *SE* = 1.41, [4.64, 10.27] | *B* = 8.24, *SE* = 1.26, [5.74, 10.75] | *B* = 8.15, *SE* = 1.71, [5.04, 11.72] | *B* = 11.39, *SE* = 2.06, [7.32, 15.41] | *B* = 12.93, *SE* = 1.49, [10.12, 15.99] |
| Total effect | 23.83*** | 18.83*** | 18.17*** | 24.58*** | 27.86*** | 25.55*** |
| **Detachment** | | | | | | |
| Direct effect | 14.20*** | 8.76*** | 6.55*** | 16.49*** | 12.87*** | 10.49*** |
| Indirect effect | *B* = 8.53, *SE* = 1.44, [5.86, 11.40] | *B* = 8.90, *SE* = 1.73, [5.78, 12.48] | *B* = 10.09, *SE* = 1.60, [7.18, 13.48] | *B* = 8.48, *SE* = 1.65, [5.34, 11.86] | *B* = 13.49, *SE* = 2.26, [9.39, 18.19] | *B* = 14.37, *SE* = 2.69, [11.15, 17.92] |
| Total effect | 22.73*** | 17.66*** | 16.65*** | 24.97*** | 26.36*** | 24.86*** |
| **Antagonism** | | | | | | |
| Direct effect | 7.87*** | 7.36** | 6.48** | 10.01*** | 15.04*** | 10.44*** |
| Indirect effect | *B* = 12.44, *SE* = 1.78, [9.12, 16.15] | *B* = 10.40, *SE* = 1.75, [6.99, 13.90] | *B* = 10.99, *SE* = 1.65, [7.96, 14.36] | *B* = 12.54, *SE* = 2.03, [8.78, 16.66] | *B* = 13.83, *SE* = 2.46, [9.14, 18.74] | *B* = 15.67, *SE* = 2.12, [11.65, 20.15] |
| Total effect | 20.31*** | 17.76*** | 17.48*** | 22.55*** | 28.87*** | 26.11*** |
| **Disinhibition** | | | | | | |
| Direct effect | 14.40*** | 7.70*** | 9.57*** | 11.39*** | 15.79*** | 16.43*** |
| Indirect effect | *B* = 5.98, *SE* = 1.18, [3.67, 8.38] | *B* = 7.64, *SE* = 1.62, [4.60, 10.96] | *B* = 6.80, *SE* = 1.25, [4.37, 9.32] | *B* = 8.51, *SE* = 1.81, [5.02, 12.14] | *B* = 9.19, *SE* = 2.19, [4.95, 13.60] | *B* = 8.65, *SE* = 1.46, [5.83, 11.67] |

| | | | | | | |
|---|---|---|---|---|---|---|
| Total effect | 20.38*** | 15.34*** | 16.37*** | 19.90*** | 24.98*** | 25.09*** |
| **Psychoticism** | | | | | | |
| Direct effect | 13.54*** | 8.79*** | 7.09*** | 13.06*** | 10.51*** | 9.66*** |
| Indirect effect | *B* = 7.17, *SE* = 1.10, [5.04, 9.33] | *B* = 7.36, *SE* = 1.35, [4.75, 10.06] | *B* = 8.24, *SE* = 1.23, [5.99, 10.82] | *B* = 8.23, *SE* = 1.51, [5.33, 11.26] | *B* = 12.19, *SE* = 2.05, [8.29, 16.31] | *B* = 12.36, *SE* = 1.47, [9.60, 15.34] |
| Total effect | 20.71*** | 16.14*** | 15.33*** | 21.30*** | 22.69*** | 22.02*** |

Note. Based on 5000 bootstrap samples and 95% confidence intervals, ** $p < .01$, *** $p < .001$, *B* = unstandardized beta, *SE* = standard error
